# New NICE guidance on acne vulgaris: implications for first-line management in primary care

**DOI:** 10.3399/bjgp21X717977

**Published:** 2021-11-26

**Authors:** Jane Wilcock, Laura Kuznetsov, Jane Ravenscroft, Mohammed Irfan Rafiq, Eugene Healy, Guideline Committee

**Affiliations:** Silverdale Medical Practice, Swinton, Manchester.; National Guideline Alliance, Royal College of Obstetricians and Gynaecologists, London.; Queen’s Medical Centre, Nottingham.; Pembroke Surgery, Reading.; University of Southampton, Southampton, and Honorary Consultant Dermatologist, University Hospital Southampton NHS Foundation Trust, Southampton General Hospital, Southampton.

## INTRODUCTION

*Acne Vulgaris: Management*, published on 25 June 2021, is a National Institute for Health and Care Excellence (NICE) guideline for patients with acne, their families and carers, their healthcare professionals, and commissioners.^1^ It does not consider neonatal acne. This article highlights parts of the guideline that cover skin care advice and first-line medication options in the management of acne.

The guideline also covers further areas such as ‘Referral to specialist care’, ‘Oral isotretinoin’, Physical treatment’, ‘Relapse’, Maintenance treatment’, and ‘Management of acne-related scarring’, which will not be covered in this article.

Acne vulgaris is one of the commonest inflammatory skin conditions, reported to create 3.5 million general practice consultations per year in the UK.^2^ It usually affects adolescents and young adults, and can last for several years. Acne vulgaris most commonly affects the face but chest, back, and shoulders may be involved. Lesions are a combination of non-inflammatory (comedones) and inflammatory lesions (papules, pustules, cysts, nodules), often leading to scarring. The committee agreed to grade acne vulgaris into ‘Mild to Moderate’ or ‘Moderate to Severe’ in order to conduct separate network meta-analysis, while accepting that acne lies along a continuum. This is easily accomplished in busy clinics and provides a baseline for assessing treatment response.

Mild-to-moderate acne vulgaris includes patients who have one or more of:

- any number of non-inflammatory lesions (comedones);
- up to 34 inflammatory lesions (with or without non-inflammatory lesions); or
- up to two nodules.

Moderate-to-severe acne vulgaris includes patients who have either or both of:

- 35 or more inflammatory lesions (with or without non-inflammatory lesions); or
- three or more nodules.

## WHAT ARE THE AIMS OF THERAPY?

Acne vulgaris is treated to prevent the development of new lesions (while allowing the older lesions to resolve) and also to prevent irreversible scarring and negative impacts on psychosocial morbidity. It is recognised that some patients with acne vulgaris may develop, or suffer a deterioration in, mental health conditions including anxiety and depression due to their skin disease. Patients should be offered information and support. Attention is given to reducing harms, including mental health deterioration and antimicrobial resistance. There was not enough evidence to support specific dietary advice for patients with acne. In deciding treatment, special populations were also considered, and flexibility given to tailor decision making and treatment to particular circumstances. This includes women who are wanting to conceive or are pregnant, as topical and oral retinoids are contraindicated in pregnancy.^3^^,^^4^

## WHAT SKIN ADVICE SHOULD BE OFFERED?

The guideline advises patients with acne to use a non-alkaline synthetic detergent (syndet) cleansing product twice daily on acne-prone skin. Syndet is a blend of synthetic surfactants, formulated to have a neutral to slightly acidic pH and high fatty acid concentration, which is less irritant than traditional soap, helps hydrate the skin, and rinses off easily. Syndets are widely available in solid and liquid form as skin-cleansing products. The limited evidence available suggests that syndet skin-cleansing products used twice daily reduce inflammatory and non-inflammatory acne vulgaris lesion counts.

Patients with acne who use skincare products, for example, moisturisers and sunscreens, are advised to avoid oil-based and comedogenic preparations. Patients with acne who use make-up are likewise advised to avoid oil-based and comedogenic products, and to remove make-up at the end of the day. Avoiding picking or scratching of acne lesions may reduce the risk of scarring.

## WHAT ARE THE OPTIONS FOR STARTING TREATMENT?

Table 1 lists the recommendations for a 12-week course of one of the first-line treatment options, taking into account the severity of the person’s acne and their preferences, and after a discussion of the advantages and disadvantages of each option. While these options are suitable for most patients, there is additional comment on alternatives for those unable to tolerate, or with contraindications, or wishing to avoid any of these products. Twelve weeks for the initial treatment duration was agreed on by the committee because positive effects can take 6–8 weeks to become noticeable and analysed studies tended to report at 12 weeks minimum.

**Table 1.** Treatment choices for mild-to-moderate and moderate-to-severe acne vulgaris

**Acne severity**	**Treatment**	**Advantages**	**Disadvantages**
Any severity	Fixed combination of topical adapalene with topical benzoyl peroxide, applied once daily in the evening	Topical Does not contain antibiotics	Not for use during pregnancy and with caution during breastfeeding (see recommendation 1.5.8) Can cause skin irritation (see recommendation 1.5.7), photosensitivity, and bleaching of hair and fabrics
Any severity	Fixed combination of topical tretinoin with topical clindamycin, applied once daily in the evening	Topical	Not for use during pregnancy or breastfeeding (see recommendation 1.5.8) Can cause skin irritation (see recommendation 1.5.7) and photosensitivity
Mild to moderate	Fixed combination of topical benzoyl peroxide with topical clindamycin, applied once daily in the evening	Topical Can be used with caution during pregnancy	Can cause skin irritation (see recommendation 1.5.7), photosensitivity, and bleaching of hair and fabrics
Moderate to severe	Fixed combination of topical adapalene with topical benzoyl peroxide, applied once daily in the evening, plus either oral lymecycline or oral doxycycline taken once daily	Oral component may be effective in treating affected areas that are difficult to reach with topical treatment (such as the back) Treatment with adequate courses of standard therapy with systemic antibiotics and topical therapy is an MHRA requirement for subsequent oral isotretinoin (see recommendation 1.5.8 and the MHRA alert on *Isotretinoin for Severe Acne: Uses and Effects*)^4^	Not for use in pregnancy, during breastfeeding or under the age of 12 (see recommendation 1.5.8) Topical adapalene and topical benzoyl peroxide can cause skin irritation (see recommendation 1.5.7), photosensitivity, and bleaching of hair and fabrics Oral antibiotics may cause systemic side effects and antimicrobial resistance Oral tetracyclines can cause photosensitivity
Moderate to severe	Topical azelaic acid applied twice daily, plus either oral lymecycline or oral doxycycline taken once daily	Oral component may be effective in treating affected areas that are difficult to reach with topical treatment (such as the back) Treatment with adequate courses of standard therapy with systemic antibiotics and topical therapy is an MHRA requirement for subsequent oral isotretinoin (see recommendation 1.5.8 and the MHRA alert on *Isotretinoin for Severe Acne: Uses and Effects*)^4^	Not for use in pregnancy, during breastfeeding, or under the age of 12 (see recommendation 1.5.8) Oral antibiotics may cause systemic side effects and resistance Oral tetracyclines can cause photosensitivity

*©NICE 2021.* Acne vulgaris: management. NG198. *Available from https://www.nice.org.uk/guidance/ng198. All rights reserved. Subject to Notice of rights. NICE guidance is prepared for the National Health Service in England. All NICE guidance is subject to regular review and may be updated or withdrawn. NICE accepts no responsibility for the use of its content in this product/publication. MHRA = Medicines and Healthcare products Regulatory Agency. NICE = National Institute for Health and Care Excellence.*

To reduce risks of skin irritation associated with topical treatments, such as benzoyl peroxide or retinoids, it is recommended to start with an alternate-day or short-contact application (for example, washing off after an hour). If tolerated, the patient can progress to using a standard application.

Monotherapy with a topical antibiotic, monotherapy with an oral antibiotic, or using a combination of topical antibiotic and an oral antibiotic are not recommended because of concerns about promotion of antibiotic resistance in conjunction with lower clinical and cost-effectiveness of oral antibiotics when used as monotherapy in moderate-to-severe acne, and no clinical effectiveness in mild-to-moderate acne. Monotherapy with benzoyl peroxide is suggested outwith the first-line treatment table for patients who wish to avoid topical retinoids or antibiotics, or in whom these agents are contraindicated.

## CONCLUSION

This guideline provides a variety of options for primary care clinicians to consider when discussing management with patients with acne vulgaris. Exploring social and psychological impacts, individualising treatment, and avoiding risks of antibacterial resistance from long-term use of antibiotics are key to improving care. The guidance recommends a range of first-line treatments using topical combined therapies, including information regarding safety in pregnancy. Advice on application should improve tolerability. When oral antibiotics are merited, an initial 3-month course should be offered with topical therapy, either topical combined retinoid and benzoyl peroxide or azelaic acid, to ensure optimal treatment without promoting antimicrobial resistance. This adheres to Medicines and Healthcare products Regulatory Agency (MHRA) guidelines for patients where subsequent oral retinoids may be required, as previous use of antibiotics should be tried.^5^

Structured follow-up 12 weeks after therapy initiation is recommended to monitor treatment response and discuss options for ongoing management. Clinicians who wish to better understand treatment options and pathways for this common and distressing condition across primary and secondary care are strongly advised to read the guideline in full.
